# TESTING A NEW MODEL OF REABLEMENT-FOCUSED INTEGRATED CARE IN ADULT DAY SERVICE USERS IN TAIWAN: A PRELIMINARY STUDY

**DOI:** 10.1093/geroni/igac059.2903

**Published:** 2022-12-20

**Authors:** Yu-Ting Chang, Hsiao-Wei Yu, Pay-Shin Lin

**Affiliations:** Chang Gung University, Taoyuan, Taoyuan, Taiwan (Republic of China); Chang Gung University of Science and Technology, Taoyuan City, Taoyuan, Taiwan (Republic of China); Chang Gung University, Taoyuan, Taoyuan, Taiwan (Republic of China)

## Abstract

There is a growing consensus that implementing personal-centered therapeutic activities at adult day services (ADS) leads to functional improvement in care recipients and reduces the burden on caregivers. Taiwan has introduced reablement-focused integrated care (RFIC) model for ADS users, the effects of this new model merit investigation. This study aimed to examine whether the RFIC model promotes physical and mental function in care recipients and improves caregiver satisfaction. We recruited ADS-care recipients and their family caregivers (N = 14 dyads) through purposive sampling for a quasiexperimental study. Six dyads were assigned to the RFIC intervention group and eight dyads to the control group. The RFIC intervention consisted of ten one-hour sessions over two months. The functional abilities of care recipients and the care satisfaction of caregivers were assessed at baseline and after the intervention. The RFIC group, compared to the control group, showed an improvement in care satisfaction score, and the group difference was significant (RFIC group: mean (SD) change score = 1.17 (2.64); control group: mean (SD) change score = −3.38 (2.20); p < 0.01). There were no differences in physical and mental function between the RFIC and control groups. These preliminary results suggest that when reablement is used as a means of personal-centered therapeutic training for ADS users, it can help caregivers cope with care problems and improve how they appraise their mastery and satisfaction as regards care. However, the effects of RFIC on the physical and mental function of care recipients require further investigation.

